# Young-Onset Early Colorectal Cancer Had Similar Relative Survival to but Better Overall Survival Than Conventional Early Colorectal Cancer: A Large Population-Based Study

**DOI:** 10.3389/fonc.2020.00096

**Published:** 2020-02-27

**Authors:** Jin-Nan Chen, Qing-Wei Zhang, Yuan-Bo Pan, Qi-Wen Wang, Xin-Tian Zhang, Xiao-Bo Li

**Affiliations:** ^1^Key Laboratory of Gastroenterology and Hepatology, Division of Gastroenterology and Hepatology, Ministry of Health, School of Medicine, Shanghai Institute of Digestive Disease, Renji Hospital, Shanghai Jiao Tong University, Shanghai, China; ^2^Department of Neurosurgery, Second Affiliated Hospital, School of Medicine, Zhejiang University, Hangzhou, China

**Keywords:** early colorectal cancer, young-onset, prognosis, cause-specific survival, relative survival

## Abstract

**Background:** There existed limited evidence about prognosis of young-onset early colorectal cancer (ECRC). In the present study, we aimed to compare prognosis between patients with young-onset ECRCs and patients with conventional ECRCs.

**Method:** Patients with surgically resected, histologically diagnosed ECRCs were retrieved from the Surveillance, Epidemiology, and End Results (SEER) database. Young-onset ECRC was defined as ECRC occurring in patients aged <50 years. Five-years relative survival was calculated at the time of diagnosed year and linear regression was performed to analyze the association between 5-years relative survival and age. The multivariate Cox regression, multivariate competing risk model, and propensity score matching (PSM) and univariate analysis weighted by the inverse probability of treatment weight (IPTW) were used to compare overall survival (OS) between young-onset ECRCs and conventional ECRCs.

**Results:** A total of 51,197 ECRCs were retrieved from SEER database, including 4,634 young-onset ECRCs and 46,563 conventional ECRCs. Five-years relative survival was found to be moderately associated with different age groups (*R* = −0.725, *P* = 0.0034). Patients with young-onset ECRCs (96.7%) had similar 5-years relative survival compared with conventional ECRCs (96.3%). However, multivariate Cox regression [HR (hazard ratio), 0.18; 95% CI: 0.16–0.20; *P* < 0.001] showed better OS in young-onset ECRCs. After PSM, we still found favored prognosis for young-onset ECRCs under univariate Cox regression (HR, 0.18; 95% CI: 0.16–0.21; *P* < 0.001). Similar results could also be found in the univariate Cox regression weighted by IPTW (HR, 0.17; 95% CI: 0.17–0.18; *P* < 0.001).

**Conclusions:** Patients with young-onset ECRCs had similar relative survival but better OS compared with conventional ECRCs.

## Introduction

According to the newest global cancer statistics, colorectal cancer (CRC) is considered the second most common cause of cancer-related death worldwide (1). Although it is well-established that the incidence of CRC increases with age, with ~90% CRCs occurring in individuals over 50 years old (2), recent studies showed increased incidence trend of CRC in young-onset groups (3–5). This increased incidence of CRC in young individuals has been observed due to effective screening for CRC in individuals under 50 years old (3–5).

Limited evidence has shown unique etiology and biology of young-onset CRCs (6, 7). Young-onset CRC presents at more advanced stage and with more aggressive clinicopathological characteristics (3–5). Meanwhile, young-onset CRCs have shown higher rate of NRAS and PTEN mutations and less rate of BRAF mutation (8). Due to the abovementioned difference in etiology and biology for young-onset CRCs, multiple studies have explored whether more attention is needed for management of young-onset CRCs with controversy results (9–15). Some studies showed favored survival in young-onset CRCs even with more advanced stage and more aggressive behavior (2, 9, 10, 14), while some studies found equivalent survival or even poorer survival for young-onset CRCs (10, 11, 15).

Early CRC (ECRC) is defined as CRC confined in the mucosa or submucosa regardless of lymph node metastasis (LNM). With advanced development of endoscopic technology, some cases of ECRCs without LNM could be managed with endoscopic resection. Although controversial conclusions about survival of young-onset CRCs were demonstrated, it was demonstrated that young-onset CRCs at early stage may have equivalent survival or even better survival than conventional CRCs (9, 10, 13, 16), indicating that young-onset ECRCs could be managed the same way as conventional ECRCs. However, no study to date have ever been performed to comprehensively analyze whether young-onset ECRCs really have better survival than conventional ECRCs in large population diagnosed ECRCs.

In this study, we would analyze whether there existed difference in cause-specific survival (CSS) and overall survival (OS) between young-onset ECRCs and conventional ECRCs in a large population. Relative survival is also used to adjust for changes in survival in population. Meanwhile, additional propensity score matching (PSM) and the inverse probability of treatment weight (IPTW) would be used to adjust for potential confounding factors.

## Methods

### Patients

In this study, using private Surveillance, Epidemiology, and End Results (SEER) ID (zhangqw), we included patients with ECRCs from the SEER database. Since the SEER database is a publicly available database, no informed consent from patients, or institutional review was required for this study.

In the present study, ECRC was defined as CRC confined to the mucosa or submucosa regardless of LNM. Patients included in our study should meet the following criteria: (1) Patients aged 18 or more who were diagnosed as ECRC between 1988 and 2015; (2) ECRC was the first diagnosed primary tumor based on The Third Edition of International Classification of Diseases for Oncology (ICD-O-3), patients who diagnosed with colon cancer (C18.0, C18.1, C18.2, C18.3, C18.4, C18.5, C18.6, C18.7, C18.8, C18.9) and rectum cancer (C19.9, C20.9); (3) surgery was performed for histologically confirmed ECRC; (4) the number of retrieved lymph nodes was available; (5) ECRC was histologically confirmed to be colorectal adenocarcinoma; and (6) patients received no preoperative radiotherapy.

Since most guidelines recommend CRC screening begin in average-risk population aged ≥50 years old (17–19) and it is well-established in the literature that 90% of CRCs occurred in patients over 50 years old (2), we defined ECRC occurring in patients aged <50 years old as young-onset ECRC and ECRC occurring in patients aged ≥50 years old as conventional ECRC. Considering there existed a different definition for young-onset CRC with cutoff age of 50 years (9, 12, 13) or 45 years (11, 14) or 40 years (10, 20), we also did sensitivity analyses in population using 45 and 40 years as cutoff age for young-onset CRC.

### Variables and Outcomes

In the present study, ECRCs were classified as young-onset ECRC and conventional ECRC with cutoff age of 50 as definition. Race of patients was recorded as white, black, or others (mainly including American Indian, Asian, and Pacific Islander). Sex was recorded as male or female. Location of primary tumor was mainly classified into three different sites: colon of left side, colon of right side, and rectum. Colon of right side consisted of appendix, cecum, ascending colon, and hepatic flexure, and colon of left side consisted of transverse colon, splenic flexure, descending colon, and sigmoid colon. Rectum consisted of rectum and rectosigmoid junction. In this study, grade I (well-differentiated) and grade II (moderately differentiated) were recoded as low grade and grade III (poorly differentiated) and grade IV (undifferentiated) were recoded as high grade. With respect to the tumor size, CRCs were classified into four groups: ≤2, ≤3, ≤5, and >5 cm. According to the invasion depth of ECRCs, they were coded as mucosa and submucosa. Since the number of adequate retrieved lymph nodes as 12 was recommended for staging of CRC and associated with survival of CRC, we also divided number of retrieved lymph nodes of ECRCs into two groups: 1–11 and no <12.

Survival time was defined as the time from diagnosis to the date of death or last contact or November 2016. Relative survival and OS were the primary outcome. Briefly, relative survival was defined as the ratio of observed survival rate of cancer patients to the expected survival rate of the matched general population and OS was defined as death regardless of causes. CSS was the second primary outcome that defined as death due to ECRC. When using competing risk model, death was classified into two groups: death due to ECRCs and death not related to ECRCs. Patients who were still alive were censored at the date of last contact.

### Statistical Analysis

For descriptive statistics, the absolute number with proportion for categorical variable, mean, and standard deviation for continuous variable with Gaussian distribution, and median with interquartile range (IQR) for continuous variable with non-normally distribution were used, respectively. The chi-square test for categorical variable, Student *t*-test for continuous variable with Gaussian distribution, and the non-parametric Kruskal–Wallis rank sum test for continuous variable with non-normally distributed data or ordinal categorical variable were used for comparisons among different patient groups, respectively.

The CSS was estimated by Kaplan–Meier curves, where patients who had death not related to ECRC or were still alive were considered censored. Log-rank test was used to analyze the differences between them. For competing risk model, the outcome of interest was defined as ECRC-specific death and death not related to ECRC was considered as a competing risk. The patients who were still alive were censored. Cumulative incidence function for ECRC-specific death was conducted, considering death not related to ECRC as a competing risk death. Nelson-Aalen cumulative risk curves of cumulative incidence of ECRC-specific death were also performed and Gray's test was applied.

The expected survival rate for the general population was obtained from SEER and the 5-years relative survival was estimated by using Ederer II method. Meanwhile, linear regression was performed to determine the correlation between 5-years relative survival and different age groups (21).

For extracted patients, each unknown value was imputed by a separate model based on the fully conditional specification (22). Multivariate Cox regression was performed to explore potential risk factors for poor OS or CSS and results were presented with hazard ratios (HRs) and 95% CIs. Meanwhile, Fine and Gray's competing risk regression was also used to determine the potential risk factors associated with ECRC-specific death, with results presented by sub-distribution hazard ratios (SHRs) and 95% CIs.

To adjust for potential imbalance between young-onset ECRCs and conventional ECRCs, we used PSM to get a new database with matched ECRCs for analysis. PSM was performed as follows: first, propensity scores were calculated for every patient with ECRCs using CRC type (young-onset ECRC or conventional ECRC) as the outcome in the multivariate logistic regression model and all potential confounding factors in Table 1 as covariates. Second, we matched young-onset ECRCs with conventional ECRCs using 1:1 matching criterion with a caliper of 0.05. Third, we calculated standard difference (SD) of all clinical characteristics between young-onset ECRCs and conventional ECRCs with a cutoff of SD as 0.1 indicating well-balanced, indicating that these clinical characteristics between young-onset ECRCs and conventional ECRCs were well-balanced. Fourth, we performed the Kaplan–Meier survival analyses with log-rank test and Nelson–Aalen cumulative risk curves with Gray's test to compare OS or CSS between young-onset ECRCs with conventional ECRCs. Besides, we also calculated HR or SHR of ECRC type on OS or CSS using univariate Cox regression model or univariate Gray's competing risk regression model.

**Table 1:** Baseline characteristics between conventional ECRC and young-onset ECRC groups with standardized difference before and after matching.

**Characteristics**	**Before matching**			**After matching**		
	**Conventional ECRC**	**Young-onset ECRC**	**SD**	**Conventional ECRC**	**Young-onset ECRC**	**SD**
	***N* = 46,563**	***N* = 4,634**		***N* = 4,634**	***N* = 4,634**	
**Race**			7.4			1.4
White	37,491 (0.8052)	3605 (0.7779)		3580 (0.7726)	3605 (0.7779)	
Black	5136 (0.1103)	617 (0.1331)		626 (0.1351)	617 (0.1331)	
Others	3936 (0.0845)	412 (0.0889)		428 (0.0924)	412 (0.0889)	
**Sex**			6.2			1.7
Female	22,876 (0.4913)	2420 (0.5222)		2381 (0.5138)	2420 (0.5222)	
Male	23,687 (0.5087)	2214 (0.4778)		2253 (0.4862)	2214 (0.4778)	
**Primary site**			44.8			1.2
Left side	19,007 (0.4082)	2220 (0.4791)		2213 (0.4776)	2220 (0.4791)	
Right side	17,489 (0.3756)	861 (0.1858)		883 (0.1905)	861 (0.1858)	
Rectum	10,067 (0.2162)	1553 (0.3351)		1538 (0.3319)	1553 (0.3351)	
**Grade**			4			0.7
Low grade	42,629 (0.9155)	4189 (0.904)		4199 (0.9061)	4189 (0.904)	
High grade	3934 (0.0845)	445 (0.096)		435 (0.0939)	445 (0.096)	
**Histology**			3.3			0.7
Conventional adenocarcinoma	45,286 (0.9726)	4490 (0.9689)		4488 (0.9685)	4490 (0.9689)	
Mucinous adenocarcinoma	1181 (0.0254)	127 (0.0274)		127 (0.0274)	127 (0.0274)	
Signet cell carcinoma	96 (0.0021)	17 (0.0037)		19 (0.0041)	17 (0.0037)	
**LNM**			16.6			1.0
No	42,888 (0.9211)	4034 (0.8705)		4018 (0.8671)	4034 (0.8705)	
Yes	3675 (0.0789)	600 (0.1295)		616 (0.1329)	600 (0.1295)	
N1a	2177 (0.0468)	342 (0.0738)		357 (0.077)	342 (0.0738)	
N1b	1129 (0.0242)	183 (0.0395)		168 (0.0363)	183 (0.0395)	
N2a	277 (0.0059)	54 (0.0117)		65 (0.014)	54 (0.0117)	
N2b	92 (0.002)	21 (0.0045)		26 (0.0056)	21 (0.0045)	
**Examined lymph node**			18.1			1.5
≤ 12	24,484 (0.5258)	2019 (0.4357)		1984 (0.4281)	2019 (0.4357)	
>12	22,079 (0.4742)	2615 (0.5643)		2650 (0.5719)	2615 (0.5643)	
**Invasion depth**			6.3			1.9
Mucosa	11,959 (0.2568)	1065 (0.2298)		1103 (0.238)	1065 (0.2298)	
Submucosa	34,604 (0.7432)	3569 (0.7702)		3531 (0.762)	3569 (0.7702)	
**Size**			2.2			2.2
≤ 2	16,389 (0.352)	1627 (0.3511)		1606 (0.3466)	1627 (0.3511)	
≤ 3	12,783 (0.2745)	1252 (0.2702)		1290 (0.2784)	1252 (0.2702)	
≤ 5	12,362 (0.2655)	1224 (0.2641)		1196 (0.2581)	1224 (0.2641)	
> 5	5029 (0.108)	531 (0.1146)		542 (0.117)	531 (0.1146)	
**Follow-up time (Median, IQR)**	76 (34,128)	93 (46,154.75)		75 (33,127)	93 (46,154.75)	

*ECRC, early colorectal cancer; SD, standardized difference; LNM, lymph node metastasis*.

IPTW is a way that allows one to obtain unbiased estimates of average treatment effects based on propensity score. Compared with PSM, IPTW can conduct data statistics without loss of sample size. Based on propensity score, we calculated the IPTW for each patient (23). SD of all clinical characteristics between young-onset ECRCs and conventional ECRCs were calculated after weighting the full cohort by IPTW with <0.1 indicating well-balanced. The unmatched univariable analysis based on the Kaplan–Meier estimator of IPTW was firstly performed. Meanwhile, univariate Cox regression model for CSS or OS or univariate Gray's competing risk regression model for CSS weighted by IPTW was performed to compare survival between young-onset ECRCs with conventional ECRCs.

Sensitivity analyses were also performed. We did sensitivity analyses in the unmatched population using 45 and 40 years as cutoff age for young-onset CRC since different cutoff ages were used to define young-onset CRC. We also did sensitivity analyses in the unmatched population after excluding patients older than 70 or 60 years old. To examine whether multiple imputation was proper in this study, we also compared survival between young-onset ECRCs and conventional ECRCs in the unmatched population without multiple imputation for unknown values. In the matched population using PSM, we did 1:2 or 1:3 matching or 1:1 matching in the cohort without multiple imputation for unknown values to test stability of our results.

All statistical analyses were carried out by using R statistical software (version 3.5.0) and two-sided *P* < 0.05 were considered statistically significant.

## Results

### Clinical Characteristics of Patients With ECRCs

**Figure 1:**
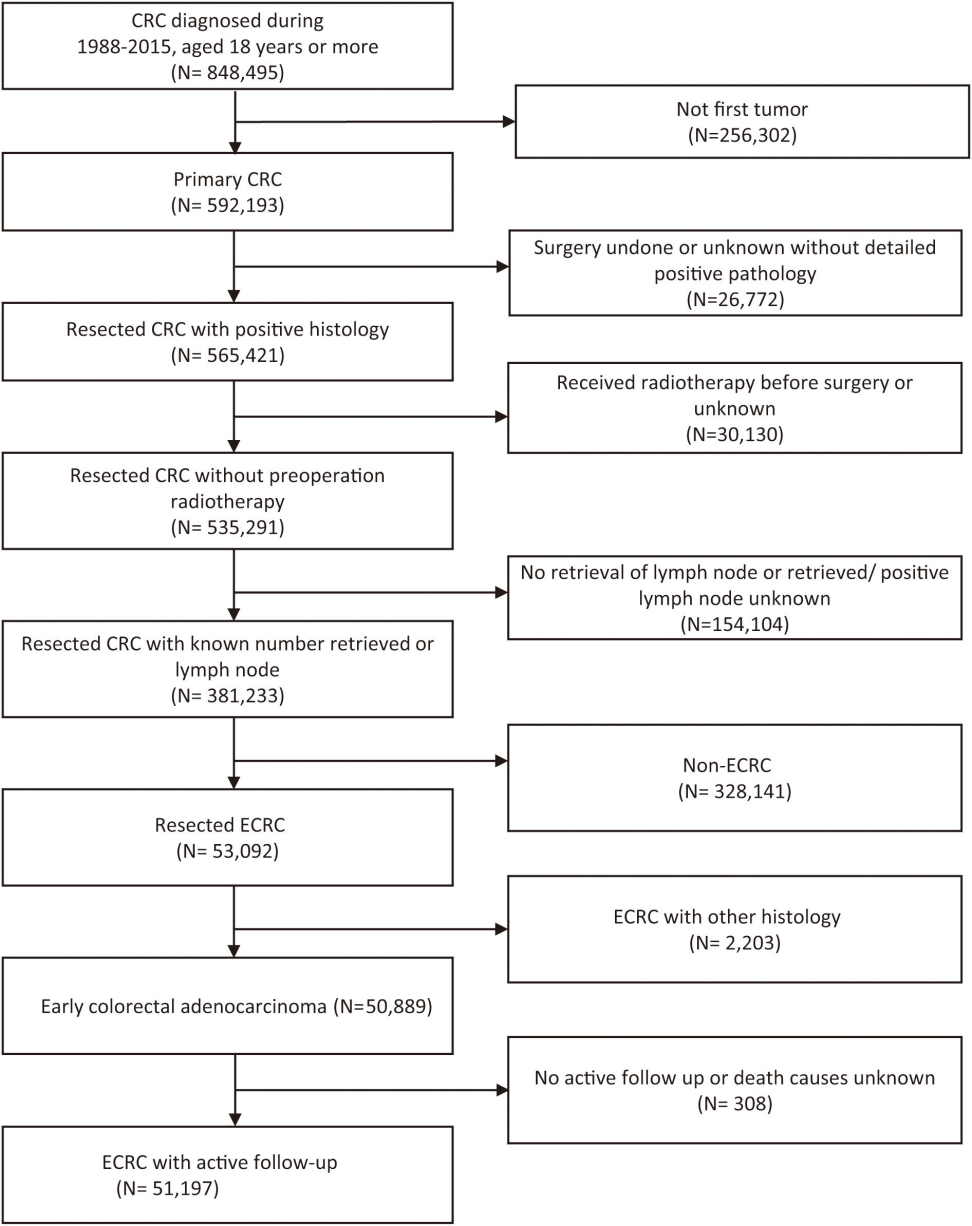
Flow chart of selection of patients with ECRC using the Surveillance, Epidemiology, and End Results database. ECRC, early colorectal cancer.

According to the inclusion and exclusion criteria (Figure 1), a total of 51,197 ECRCs were included in our study, consisting of 4,634 patients of young-onset ECRC and 46,563 patients of conventional ECRC. As is shown in Supplementary Table 1, a total of 24,100 (47.07%) ECRCs had LNM, among which young-onset ECRCs (55.03%) had higher LNM rate than conventional ECRCs (46.28%, *P* < 0.001). However, it could be seen that patients with young-onset ECRCs had longer median survival time [median: 89 (44, 148)] than patients with conventional ECRCs [median: 76 (34, 128); *P* < 0.001]. Results also demonstrated that patients with young-onset ECRCs had more lymph nodes examination than conventional ECRCs. The detailed clinical characteristics of young-onset ECRCs and conventional ECRCs were described in Supplementary Table 1 as young-onset ECRCs were more likely to occur in the left side and rectum, with higher LNM and deeper invasion depth compared with conventional ECRCs.

### Comparison of 5-Years Relative Survival Among Young-Onset ECRCs and Conventional ECRCs

We calculated the 5-years relative survival rate of different age groups of ECRCs with a 5-years interval and linear regression showed a moderate correlation (*R* = −0.725, *P* = 0.0034) between 5-years relative survival and age. We also indicated that young-onset ECRCs had a similar 5-years relative survival rate (96.7, 95% CI: 95.8–97.3) compared with conventional ECRCs (96.3, 95% CI: 95.9–96.8); the same results could be found even if we used 40 and 45 years old as cutoff points (Supplementary Figure 2 and Table 2).

**Table 2:** Five-year relative survival of young-onset ECRC and conventional ECRC.

**Age group**	**Five-year relative survival**	
	**Observed survival, % (95% CI)**	**Relative survival, % (95% CI)**
**Age** **<50 defined as young-onset ECRC**		
Young-onset ECRC	95.3 (94.5–95.9)	96.7 (95.8–97.3)
Conventional ECRC	82.7 (82.3–83.1)	96.3 (95.9–96.8)
**Age** **<45 defined as young-onset ECRC**		
Young-onset ECRC	95.9 (94.8–96.7)	96.8 (95.7–97.7)
Conventional ECRC	83.3 (82.9–83.6)	96.4 (95.9–96.8)
**Age** **<40 defined as young-onset ECRC**		
Young-onset ECRC	95.6 (93.8–96.9)	96.3 (94.5–97.5)
Conventional ECRC	83.6 (83.2–83.9)	96.4 (95.9–96.8)

### Comparison of Survival Among Young-Onset ECRCs and Conventional ECRCs in the Unmatched Cohort

**Figure 2:**
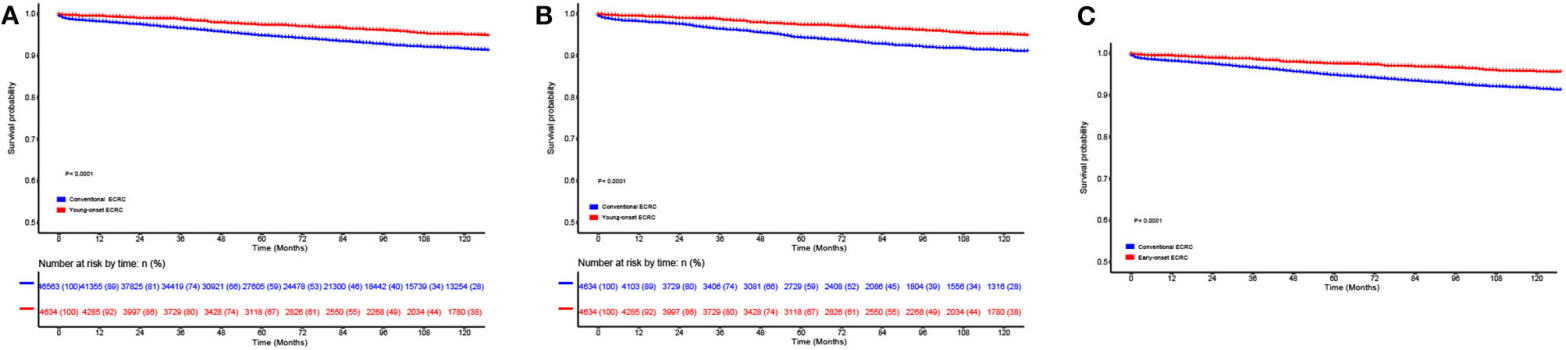
Comparison of cause-specific survival in **(A)** the unmatched, **(B)** the propensity score matched, and **(C)** the inverse probability of treatment weight–adjusted analysis between patients with conventional ECRCs and patients with young-onset ECRCs. ECRC, early colorectal cancer.

To compare survival among young-onset ECRCs and conventional ECRCs, we firstly compared 5-years CSS rate, 10-years survival rate, and CSS curves of two types. We found that patients with young-onset ECRCs had a 5-years survival rate of 97.4% and a 10-years survival rate of 95.1%, which was higher than patients with conventional ECRCs (Figure 2A, *P* < 0.001). The 5-years and 10-years survival rate of patients with conventional ECRCs were 94.8 and 91.6%, respectively. The rest of the CSS rate for different time was depicted in Supplementary Table 2.

We also calculated and compared OS rate among patients with young-onset ECRCs and patients with conventional ECRCs. As is shown in Supplementary Figure 1A and Supplementary Table 3, patients with young-onset ECRCs had higher 5 and 10-years OS rate than conventional ECRCs. The same results (Supplementary Figure 3A) could be obtained in the comparison of cumulative probability of cancer-specific death using competing risk model.

**Figure 3:**
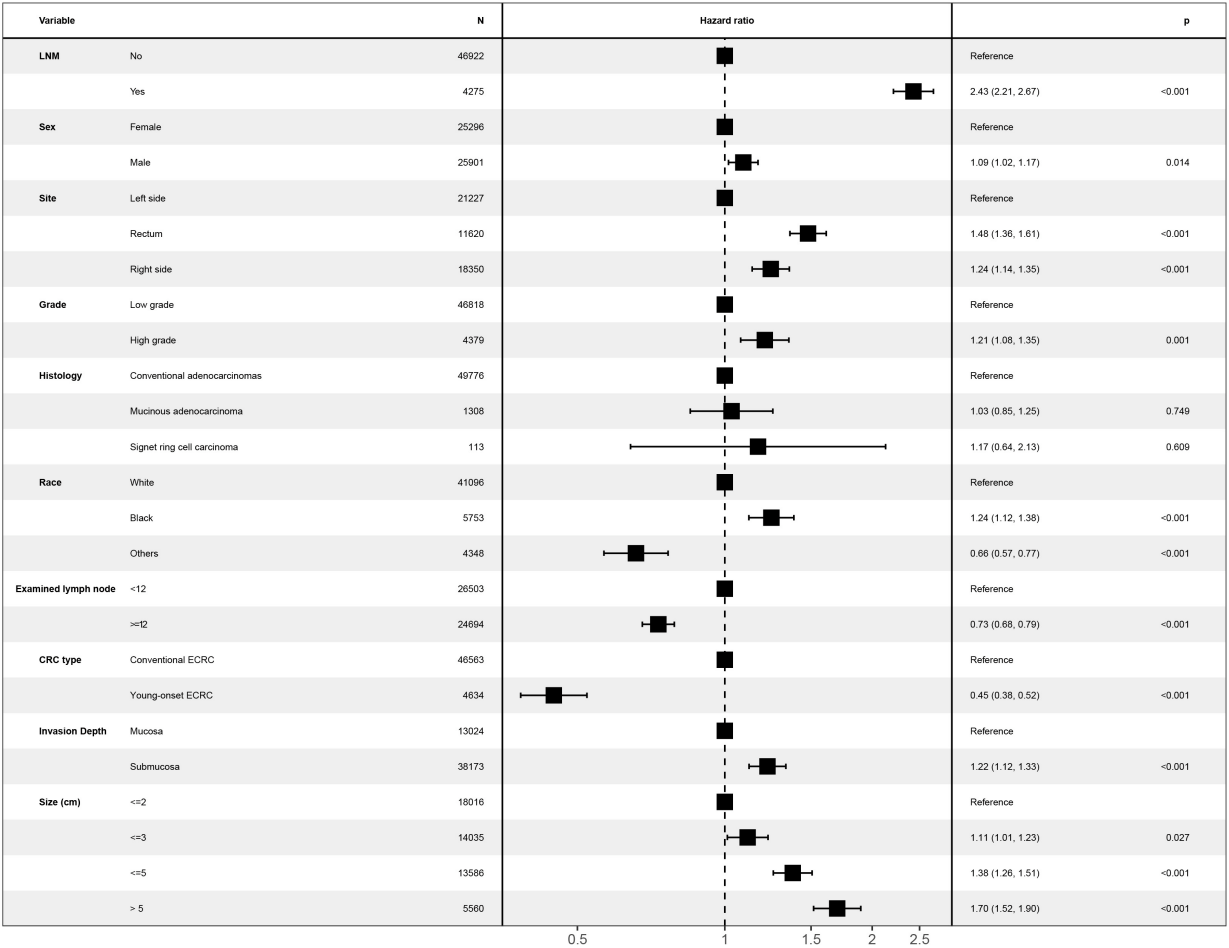
Forest plot showing results of multivariate Cox regression model for exploring potential risk factors for cause-specific survival in patients with ECRCs in 51,197 patients of the Surveillance, Epidemiology, and End Results database. LNM, lymph node metastasis; ECRC, early colorectal cancer.

Unknown values of confounding factors were interpolated using multiple imputation and clinical pathological baseline is shown in Table 1. Then, the multivariate Cox regression model was performed to investigate whether young-onset ECRCs had better CSS than conventional ECRCs after adjusting potential risk factors for CSS. As is shown in Figure 3, results showed that patients with young-onset ECRCs had better CSS than conventional ECRCs in multivariate Cox regression model (HR: 0.45, 95% CI: 0.38–0.52, *P* < 0.001). We also compared OS and CSS under competing risk model in the multivariate models and results showed that young-onset ECRCs still had better OS (Supplementary Figure 4, HR: 0.18, 95% CI: 0.16–0.20, *P* < 0.001) and lower cumulative probability of cancer-specific death (Supplementary Figure 5, SHR: 0.51, 95% CI: 0.44–0.60, *P* < 0.001) than conventional ECRCs. Furthermore, we divided ECRCs into three subtypes: left side only, right side only, and rectum only. The multivariate Cox regression was performed, respectively, to explore whether the location of ECRCs could have a different effect on CSS between these two groups. The results also demonstrated that young-onset ECRCs had better CSS than conventional ECRCs in left side type (Supplementary Table 4, HR: 0.373, 95% CI: 0.285–0.487, *P* < 0.001), right side type (Supplementary Table 5 HR: 0.488, 95% CI: 0.337–0.707, *P* < 0.001) and rectum type (Supplementary Table 6 HR: 0.504, 95% CI: 0.403–0.632, *P* < 0.001).

**Figure 4:**
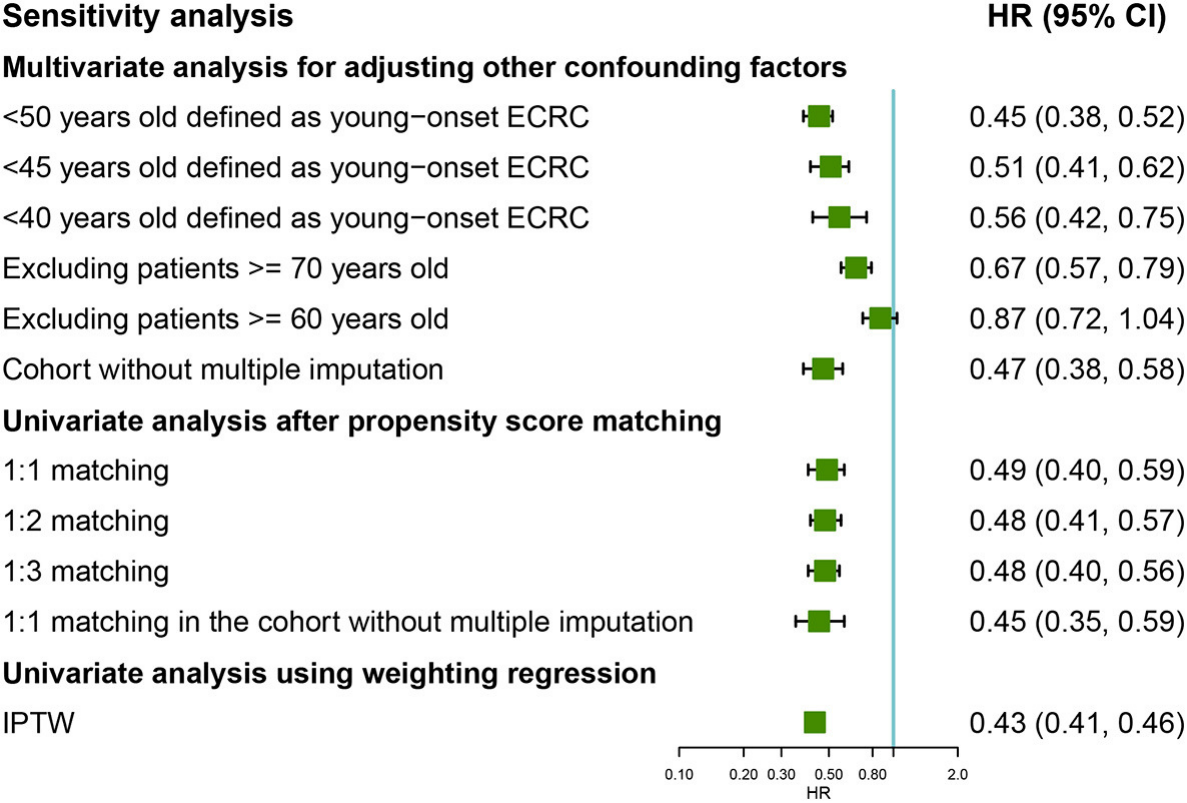
Forest plot showing results of sensitivity analyses for cause-specific survival based on different analysis strategy in the unmatched, the propensity score matched, and the inverse probability of treatment weight–adjusted analysis, respectively. LNM, lymph node metastasis; ECRC, early colorectal cancer.

To investigate whether different age cutoffs defined for young-onset ECRCs could influence CSS among these two groups, we selected 45 and 40 years old for cutoff, respectively, for sensitivity analysis. The multivariate Cox regression model (Figure 4) still supported the conclusion that patients with young-onset ECRCs had better CSS than patients with conventional ECRCs in the situation when patients aged <45 years old were defined as patients with young-onset ECRC (HR: 0.51, 95% CI: 0.41–0.62, *P* < 0.001) or patients aged <40 years old were defined as patients with young-onset ECRC (HR: 0.56, 95% CI: 0.42–0.75, *P* < 0.001). Besides, we also compared survival of two groups in patients aged <60 or aged <70. Patients with young-onset ECRCs had significantly better CSS than patients with conventional ECRCs in patients aged <70 (HR: 0.67, 95% CI: 0.57–0.79, *P* < 0.001) but without significance in patients aged <60 (HR: 0.87, 95% CI: 0.72–1.04, *P* = 0.117). Meanwhile, the multivariate Cox regression model in the cohort without multiple imputation for unknown values also identified better CSS of young-onset ECRCs than conventional ECRCs.

Using OS and CSS under competing risk model as outcome, respectively, similar sensitivity analyses identified better survival of young-onset ECRCs than conventional ECRCs (Supplementary Figures 6, 7).

### Comparison of Survival Among Young-Onset ECRCs and Conventional ECRCs in the Matched Cohort

Using PSM, we matched 4,634 young-onset ECRC patients with 4,634 conventional ECRC patients. SD calculated before and after matching showed that all confounding factors were balanced after matching (Table 1 and Supplementary Figure 8). As is shown in Supplementary Tables 2, 3, patients with young-onset ECRCs had higher CSS (Figure 2B, 5-years CSS: 97.4%, *P* < 0.001), higher OS (Supplementary Figure 1B, 5-years OS: 95.7%, *P* < 0.001), and lower cumulative probability of cancer-specific death (Supplementary Figure 3B, *P* < 0.001) than patients with conventional ECRC patients (5-years CSS: 95.0%, 5-years OS: 84.0%).

In the univariate model, patients with young-onset ECRCs were identified to favor higher CSS (HR: 0.49, 95% CI: 0.40–0.59, *P* < 0.001), higher OS (HR: 0.18, 95% CI: 0.16–0.21, *P* < 0.001), and higher CSS under competing risk model (SHR: 0.54, 95% CI: 0.45–0.65, *P* < 0.001).

The univariate model of 1:2 PSM, 1:3 PSM in the cohorts, or 1:1 PSM in the cohort without multiple imputation for unknown values still identified favored CSS, OS, or CSS under competing risk model in the young-onset ECRCs (Figure 4 and Supplementary Figures 6, 7).

### Comparison of Survival Among Young-Onset ECRCs and Conventional ECRCs Using IPTW Adjusted Analysis

Figure 2C displays the results of IPTW-adjusted analysis of CSS between patients with young-onset ECRCs and patients with conventional ECRCs. Young-onset ECRCs had higher CSS (5 years: 97.5%, 10 years: 92.2%) than conventional ECRCs (Supplementary Table 2, 5 years: 94.8%, 10 years: 91.6%, *P* < 0.001). Comparison of OS and CSS under the competing risk model in IPTW-adjusted analysis revealed similar results (Supplementary Table 3 and Supplementary Figures 1, 3).

The univariate Cox regression weighted by IPTW showed similar results that young-onset ECRCs favored better CSS (Figure 4, HR: 0.43, 95% CI: 0.41–0.46, *P* < 0.001) than conventional ECRCs. Similar results could be obtained using univariate Cox regression weighted by IPTW with OS and CSS under competing risk model as outcomes, respectively (Supplementary Figures 5, 7).

## Discussion

To the best of our knowledge, the present study was the first study to compare survival of young-onset ECRCs with conventional ECRCs comprehensively. Results of the multivariate Cox regression model showed that patients with young-onset ECRCs had better OS and better CSS than conventional ECRCs, which was consistent with results from univariate analysis after PSM or univariate Cox regression weighted by IPTW. However, patients with young-onset ECRCs and conventional ECRCs showed similar 5-years relative survival.

In this study, young-onset ECRC was defined as ECRC occurring in patients aged <50 years. It should be acknowledged that definition of young-onset CRC varied in different studies with some studies using a cutoff age of 50 years (9, 12, 13) for definition or 45 years (11, 14) or 40 years (10, 20) for definition. Though the age cutoff used in the present study was somewhat arbitrary, the cutoff of 50 was selected based on the fact that American, European, and Asian guidelines all recommended CRC screening begin in average-risk population aged ≥50 years old (17–19). Consistent with reports from most studies (2, 9–11, 13–16, 20), even diagnosed at early stage, young-onset CRC was more likely to demonstrate higher rate of LNM, submucosal invasion, and location in left-side colon or rectum (Table 1), which proved our rationality of definition for young-onset CRC to some extent. Besides, we also did sensitivity analyses by using different cutoff ages for definition for young-onset CRC and results were also consistent with our primary results.

Some studies also compared prognosis of young-onset CRCs with that of conventional CRCs in subgroups of T1 stage CRCs (9, 10, 13, 16). Studies found lower 5-years CSS (10, 13), OS (16), and disease-free survival (13) for young-onset T1 CRCs but no statistical significance were found in these studies. Only one study analyzed OS in localized CRC including T1 CRCs with results indicating that young-onset T1 CRCs may have better survival than conventional CRCs still without significant difference (9). Therefore, to explore whether young-onset T1 CRCs had comparable prognosis than conventional T1 CRCs, we performed this comprehensive analysis using multivariate regression model, univariate regression model after PSM, and univariate regression model weighted by IPTW. However, different from results found in previous studies, we found that young-onset ECRCs had higher 5-years CSS (97.4 vs. 94.8%) and OS (95.7 vs. 82.2%) than conventional ECRCs. After matching for confounding factors, better 5-years CSS (97.4 vs. 95.0%) and OS (95.7 vs. 84.0%) could be found in young-onset ECRCs, which had higher 5-years CSS (97.4 vs. 94.8%) and OS (95.7 vs. 82.2%) than conventional ECRCs. Similar 5-years CSS and OS could be obtained in the model weighted by IPTW.

In our study, 5-years relative survival rate of young-onset ECRCs and conventional ECRCs was 96.7%, which did not show significant difference compared with conventional ECRCs of 96.3%. Patients with conventional ECRCs had 5-years CSS of 94.8%, which was consistent with results reported in previous studies (10, 13). However, patients with young-onset ECRCs had 5-years CSS of 97.4%, which was a little higher than results in previous studies (10, 13). For OS, patients with young-onset ECRCs had 5-years OS of 95.7%, which was higher than results (93.3%) in a previous study (16). Five-years OS was 82.2% for conventional ECRCs, which was lower than the reported 5-years OS of 94.9% (16). Some reasons may explain our results. First, OS and CSS could not eliminate the effect of frailty of patients caused by age so that the results contradict the relative survival rate. Second, ECRC was defined as CRC confined to the mucosa or submucosa regardless of LNM. Therefore, we also included mucosal CRC (Tis) in our study, which was excluded for analysis in other studies (9, 10, 13), causing different results from other studies. Third, only colon or rectal cancers were included in all studies (10, 13, 16), but all CRCs were included for analysis in the present study, which may also cause different results.

Some limitations should be discussed in the present study. First, values of analyzed confounding factors missed for some cases, which may cause some bias in the present study. However, multiple imputation was performed to impute missed values for cases before we performed the multivariate regression model. Sensitivity analysis in the cohort without multiple imputation also showed similar conclusion to the main findings, which indicated that multiple imputation technology in the present study was reasonable. Second, due to the limited information provided in the SEER database, other outcomes including cancer recurrence and progress-free survival could be analyzed in the present study. However, cancer recurrence and progress-free survival were correlated with CSS or OS. Results of CSS or OS indicated that young-onset ECRCs may also have better cancer recurrence or progress-free survival than conventional ECRCs. Besides, the competing risk model, which takes death not related to ECRCs into consideration, was also used to assess and compare prognosis of young-onset ECRCs and conventional ECRCs. Third, some risk factors such as lymphatic vessel involvement (LVI) were missed in the present study. Actually, our aim was to compare survival between young-onset ECRCs and conventional ECRCs and LVI was a risk factor for LNM. To some extent, LNM was correlated with LVI and may not be both included in the final multivariate model.

In summary, although patients with young-onset ECRCs had higher risk of LNM, they favored better survival outcomes compared with conventional ECRCs.

## Data Availability Statement

Publicly available datasets were analyzed in this study. This data can be found here: https://seer.cancer.gov/.

## Author Contributions

X-BL and J-NC: study concept and design. J-NC, Q-WZ, and Y-BP: acquisition of data. J-NC, X-TZ, and Y-BP: analysis and interpretation of data. J-NC, Q-WZ, and X-TZ: drafting of the manuscript. X-BL: critical revision of the manuscript for important and obtained funding. J-NC and Q-WW: statistical analysis.

### Conflict of Interest

The authors declare that the research was conducted in the absence of any commercial or financial relationships that could be construed as a potential conflict of interest.
