# Supplementary material for: Young-Onset Early Colorectal Cancer Had Similar Relative Survival to but Better Overall Survival Than Conventional Early Colorectal Cancer: A Large Population-Based Study
Source: Front Oncol. 2020 Feb 27;10:96. doi: 10.3389/fonc.2020.00096 (PMC7056900; doi:10.3389/fonc.2020.00096)
Supplement: Supplementary file 1 [file Data_Sheet_1.pdf]

Supplementary Material

Appendix 1

Supplementary Table 1. Demographic and Clinical Characteristics of Patients with Colorectal Cancers

**Characteristics**	Total<br>N= 51197	Young-onset ECRC<br>N= 4634	Conventional ECRC<br>N=46563	P
**Race**				<0.001
White	40893(0.7987)	3570(0.7704)	37323(0.8016)	
Black	5724(0.1118)	615(0.1327)	5109(0.1097)	
Others	4316(0.0843)	409(0.0883)	3907(0.0839)	
Unknown	264(0.0052)	40(0.0086)	224(0.0048)	
**Sex**				0.2017
Female	25296(0.4941)	2420(0.5222)	22876(0.4913)	
Male	25901(0.5059)	2214(0.4778)	23687(0.5087)	
**Primary site**				<0.001
Left side	21019(0.4106)	2186(0.4717)	18833(0.4045)	
Right side	18151(0.3545)	848(0.183)	17303(0.3716)	
Rectum	11494(0.2245)	1527(0.3295)	9967(0.2141)	
Unknown	533(0.0104)	73(0.0158)	460(0.0099)	
**Grade**				0.0246
Low grade	36265(0.7083)	3242(0.6996)	33023(0.7092)	
High grade	3312(0.0647)	343(0.074)	2969(0.0638)	
Unknown	11620(0.227)	1049(0.2264)	10571(0.227)	
**Histology**				0.1326
Conventional adenocarcinoma	49776(0.9722)	4490(0.9689)	45286(0.9726)	
Mucinous adenocarcinoma	1308(0.0255)	127(0.0274)	1181(0.0254)	
Signet cell carcinoma	113(0.0022)	17(0.0037)	96(0.0021)	
**LNM**				<0.001
No	25732(0.5026)	1972(0.4256)	23760(0.5103)	
Yes	24100(0.4707)	2550(0.5503)	21550(0.4628)	
N1a	2503(0.0489)	340(0.0734)	2163(0.0465)	
N1b	1301(0.0254)	182(0.0393)	1119(0.024)	
N2a	327(0.0064)	52(0.0112)	275(0.0059)	
N2b	113(0.0022)	21(0.0045)	92(0.002)	
Unknown	31(6e-04)	5(0.0011)	26(6e-04)	
**Examined Lymph node**				<0.001
<=12	25732(0.5026)	1972(0.4256)	23760(0.5103)	
>12	24100(0.4707)	2550(0.5503)	21550(0.4628)	
Unknown	1365(0.0267)	112(0.0242)	1253(0.0269)	
**Depth**				<0.001
Mucosa	13024(0.2544)	1065(0.2298)	11959(0.2568)	
Submucosa	38173(0.7456)	3569(0.7702)	34604(0.7432)	
**Size**				<0.001

<=2	10973(0.2143)	939(0.2026)	10034(0.2155)	
<=3	8927(0.1744)	694(0.1498)	8233(0.1768)	
<=5	8437(0.1648)	684(0.1476)	7753(0.1665)	
>5	3355(0.0655)	318(0.0686)	3037(0.0652)	
Unknown	19505(0.381)	1999(0.4314)	17506(0.376)	
Follow-up Time (Median, IQR)	77(35,130)	89(44,148)	76(34,128)	<0.001

ECRC: early colorectal cancer; SD: standardized difference; LNM: lymph node metastasis.

Appendix 2

Supplementary Table 2. Comparison of Cause-Specific Survival Between Patients With Young-onset T1 CRC and Patients With Conventional ECRC.

Year	Unmatched		PSM		IPTW	
	Young-onset ECRC	Conventional ECRC	Young-onset ECRC	Conventional ECRC	Young-onset ECRC	Conventional ECRC
1	99.5	98.2	99.5	98.4	99.5	98.2
2	99.0	97.5	99.0	97.4	98.9	97.5
3	98.7	96.6	98.7	96.8	98.6	96.6
4	97.9	95.7	97.9	96.0	98.0	95.7
5	97.4	94.8	97.4	95.0	97.5	94.8
6	97.1	94.2	97.1	94.0	97.3	94.1
7	96.5	93.5	96.5	93.6	96.9	93.4
8	96.1	92.7	96.1	93.0	96.6	92.7
9	95.4	92.1	95.4	92.6	95.9	92.1
10	95.1	91.6	95.1	92.2	95.7	91.6

ECRC: early colorectal cancer; PSM: propensity score matching; IPTW: inverse probability of treatment weight

Appendix 3

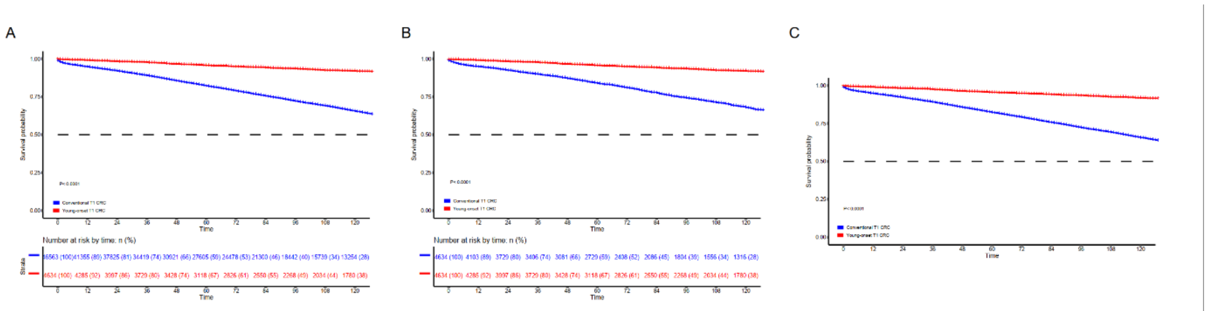

Supplementary Figure 1. Comparison of overall survival in (A) the unmatched, (B) the propensity score matched, and (C) the inverse probability of treatment weight–adjusted analysis between patients with conventional T1 CRCs and patients with young-onset T1 CRCs. CRC: colorectal cancer

Appendix 4

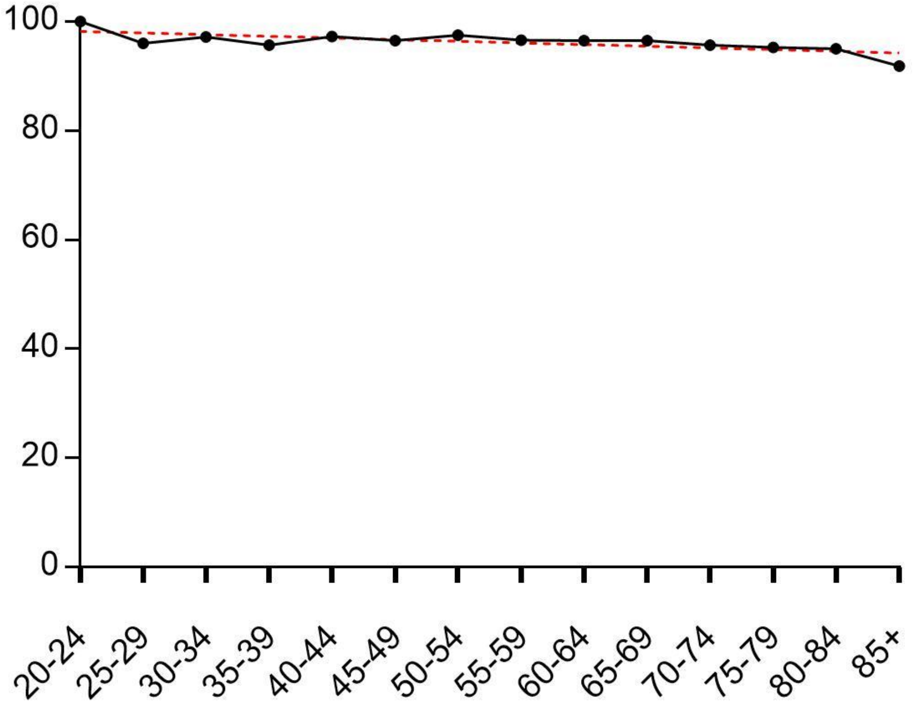

Supplementary Figure 2 relationship between 5-year relative survival (y axis) and different age groups

Appendix 5

Supplementary Table 3. Comparison of Overall Survival Between Patients With Young-onset ECRC and Patients With Conventional ECRC.

Year	Unmatched		PSM		IPTW	
	Young-onset ECRC	Conventional ECRC	Young-onset ECRC	Conventional ECRC	Young-onset ECRC	Conventional ECRC
1	99.1	94.7	99.1	95.6	99.0	94.8
2	98.3	92.0	98.3	92.7	98.2	92.1
3	97.7	89.0	97.7	90.2	97.5	89.1
4	96.6	85.6	96.6	87.0	96.4	85.7
5	95.7	82.2	95.7	84.0	95.5	82.3
6	95.0	78.9	95.0	80.1	94.9	79.0
7	94.2	75.6	94.2	76.3	94.1	75.7
8	93.6	72.1	93.6	73.6	93.5	72.2
9	92.5	69.0	92.5	71.0	92.5	69.2
10	92.0	65.5	92.0	67.6	91.8	65.7

ECRC: early colorectal cancer; PSM: propensity score matching; IPTW: inverse probability of treatment weight

Appendix 6

Supplementary Table 4. Comparison of Cause-Specific Survival Between Patients with Young-onset T1 CRC left side only and Patients with Conventional ECRC left side only.

Variables	multivariate		
	HR	95% CI	P-value
LNM Yes	2.256	1.926-2.642	<0.001
Male sex	1.104	0.983-1.240	0.096
High grade	1.175	0.964-1.432	0.110
Conventional adenocarcinoma	Reference		
Mucinous adenocarcinoma	1.078	0.758-1.534	0.677
Signet ring cell carcinoma	1.205	0.383-3.793	0.750
Race white	Reference		
Race black	1.215	1.017-1.451	0.032
Race others	0.624	0.489-0.795	<0.001
Examined lymph node>12	0.862	0.757-0.980	0.024
Young-onset CRC	0.373	0.285-0.487	<0.001
Submucosa invasion	0.976	0.843-1.131	0.750
Size level1	Reference		
Size level2	1.113	0.957-1.294	0.164
Size level3	1.454	1.255-1.684	<0.001
Size level4	1.583	1.292-1.940	<0.001

Appendix 7

Supplementary Table 5. Comparison of Cause-Specific Survival Between Patients with Young-onset T1 CRC right side only and Patients with Conventional ECRC right side only.

Variables	multivariate		
	HR	95% CI	P-value
LNM Yes	2.646	2.215-3.159	<0.001
Male sex	1.048	0.929-1.183	0.445
High grade	1.296	1.071-1.568	0.007
Conventional adenocarcinoma	Reference		
Mucinous adenocarcinoma	0.879	0.652-1.185	0.398
Signet ring cell carcinoma	0.708	0.225-2.225	0.555
Race white	Reference		
Race black	1.141	0.967-1.347	0.117
Race others	0.673	0.495-0.915	0.011
Examined lymph node<12	0.622	0.550-0.704	<0.001
Young-onset CRC	0.488	0.337-0.707	<0.001
Submucosa invasion	1.598	1.389-1.839	<0.001
Size level1	Reference		
Size level2	1.202	1.014-1.425	0.034
Size level3	1.362	1.160-1.600	<0.001
Size level4	1.761	1.471-2.108	<0.001

Appendix 8

Supplementary Table 6. Comparison of Cause-Specific Survival Between Patients with Young-onset T1 CRC rectum only and Patients with Conventional ECRC rectum only.

Variables	multivariate		
	HR	95% CI	P-value
LNM Yes	2.463	2.106-2.880	<0.001
Male sex	1.127	0.995-1.278	0.060
High grade	1.160	0.948-1.421	0.150
Conventional adenocarcinoma	Reference		
Mucinous adenocarcinoma	1.190	0.820-1.726	0.360
Signet ring cell carcinoma	1.728	0.706-4.231	0.231
Race white	Reference		
Race black	1.522	1.227-1.887	<0.001
Race others	0.700	0.546-0.896	0.005
Examined lymph node<12	0.731	0.637-0.839	<0.001
Young-onset CRC	0.504	0.403-0.632	<0.001
Submucosa invasion	1.065	0.907-1.251	0.444
Size level1	Reference		
Size level2	1.028	0.857-1.230	0.765
Size level3	1.295	1.093-1.534	0.003
Size level4	1.686	1.376-2.061	<0.001

Appendix 9

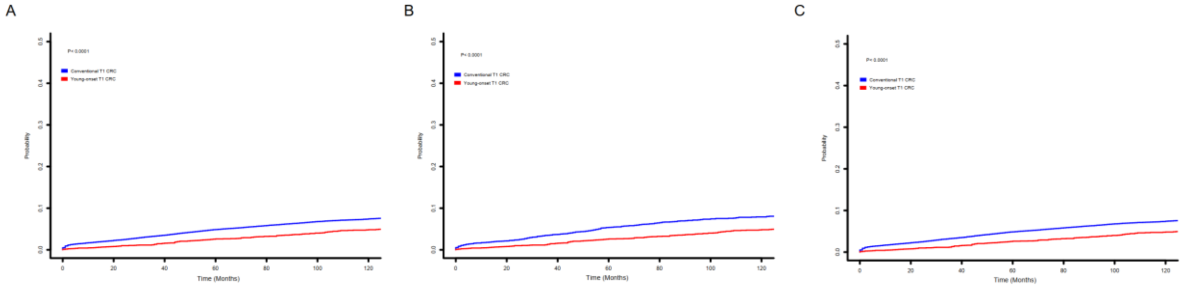

Supplementary Figure 3. Comparison of cumulative probability of cancer-specific death in (A) the unmatched, (B) the propensity score matched, and (C) the inverse probability of treatment weight–adjusted analysis using competing risk model between patients with conventional ECRCs and patients with young-onset ECRCs. ECRC: early colorectal cancer

Appendix 10

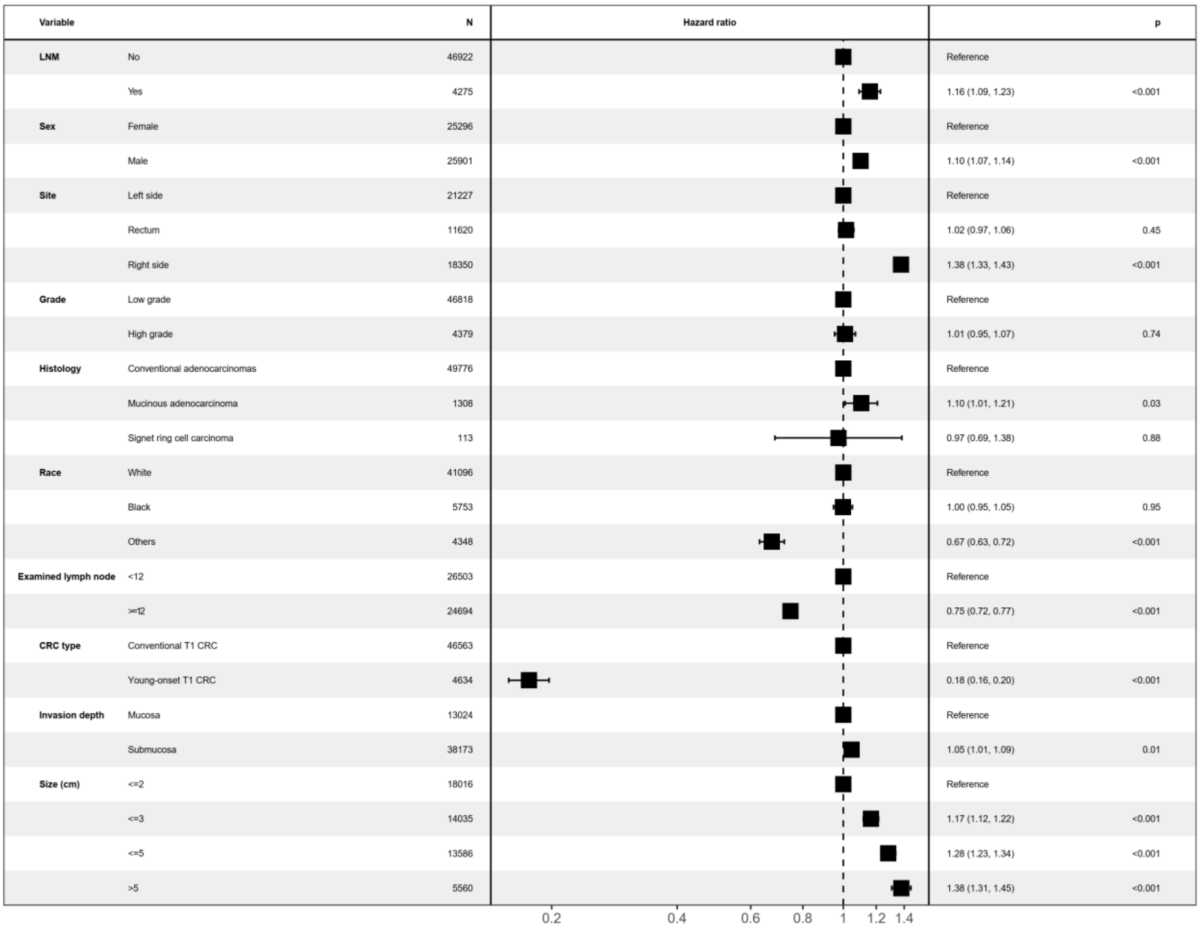

Supplementary Figure 4. Forest plot showing results of multivariate Cox regression model for exploring potential risk factors for overall survival in patients with ECRCs in 51,197 patients of the Surveillance, Epidemiology, and End Results database. LNM: lymph node metastasis; ECRC: early colorectal cancer.

Appendix 11

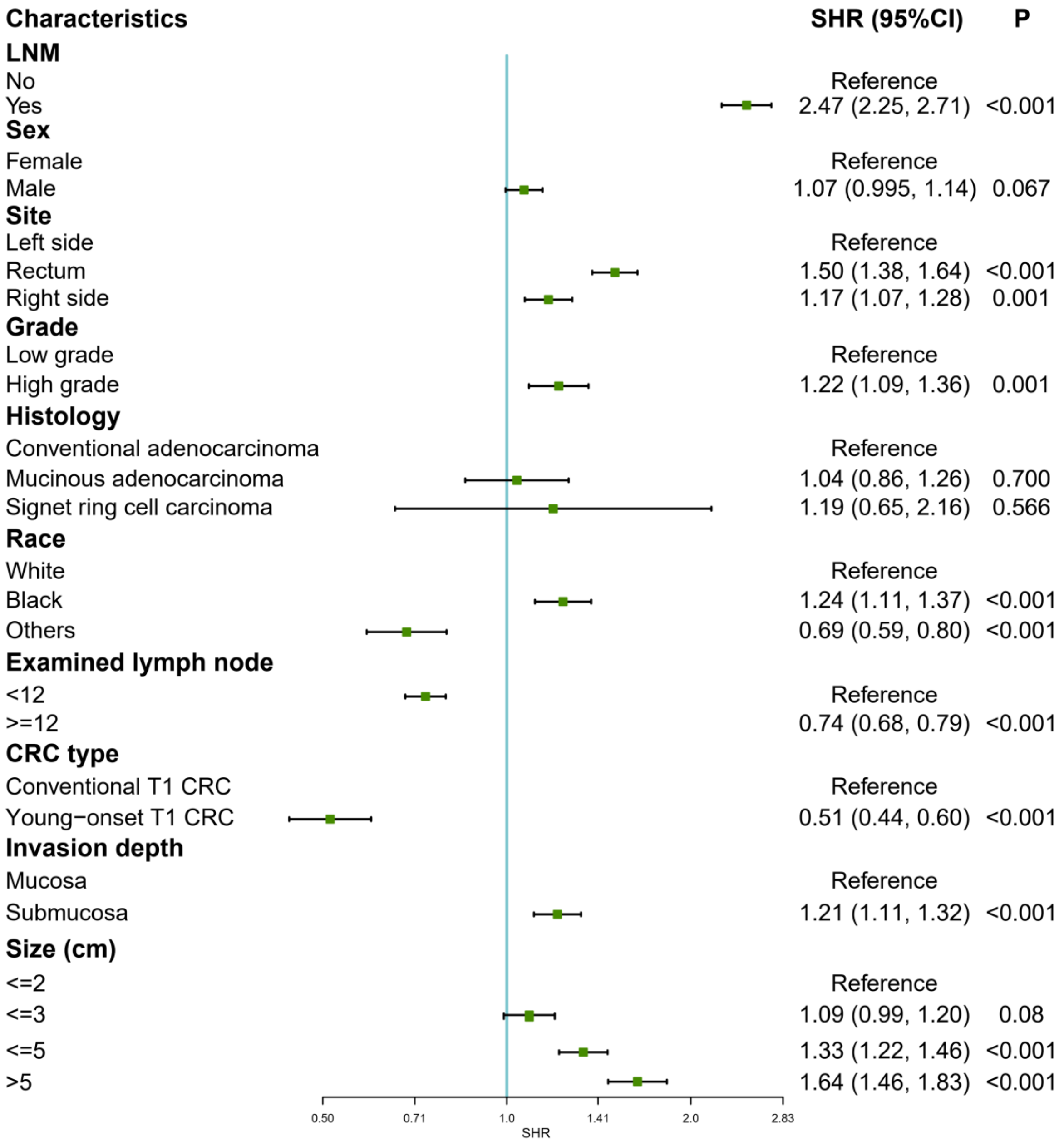

Supplementary Figure 5. Forest plot showing results of multivariate Fine-Gray regression competing risk model for exploring potential risk factors for cause-specific survival in patients with ECRC in 51,197 patients of the Surveillance, Epidemiology, and End Results database. LNM: lymph node metastasis; ECRC: early colorectal cancer. SHR: sub-distribution hazard ratio.

Appendix 12

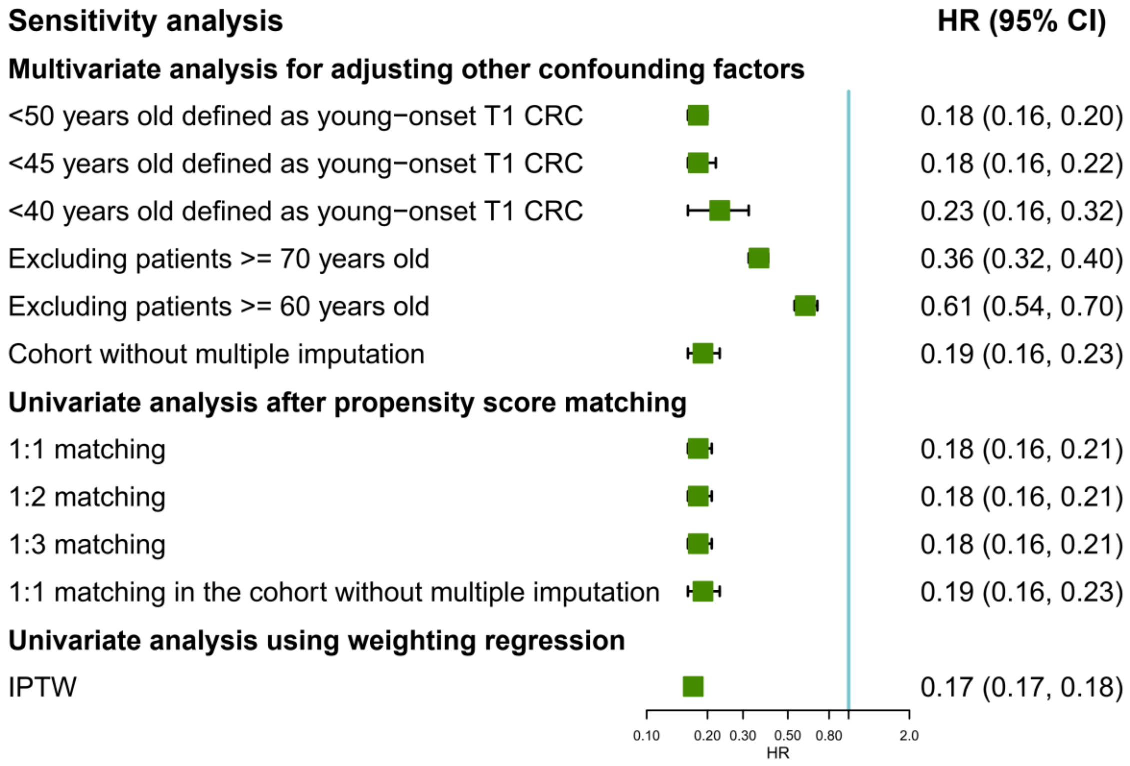

Supplementary Figure 6. Forest plot showing results of sensitivity analyses for overall survival based on different analysis strategy in the unmatched, the propensity score matched, and the inverse probability of treatment weight–adjusted analysis respectively. LNM: lymph node metastasis; ECRC: early colorectal cancer; IPTW: inverse probability of treatment weight. HR: sub-distribution hazard ratio.

Appendix 13

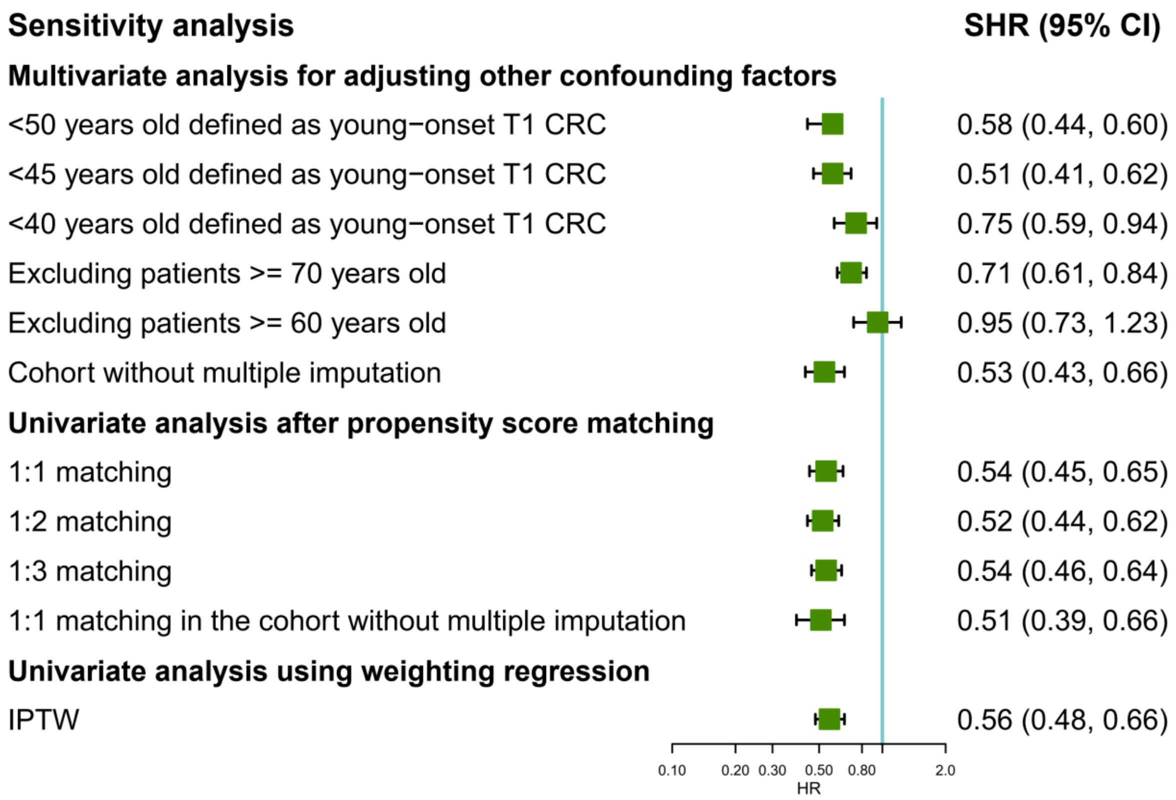

Supplementary Figure 7. Forest plot showing results of sensitivity analyses for cause-specific survival based on different analysis strategy in the unmatched, the propensity score matched, and the inverse probability of treatment weight–adjusted analysis respectively. LNM: lymph node metastasis; ECRC: early colorectal cancer; IPTW: inverse probability of treatment weight. SHR: sub-distribution hazard ratio.

Appendix 14

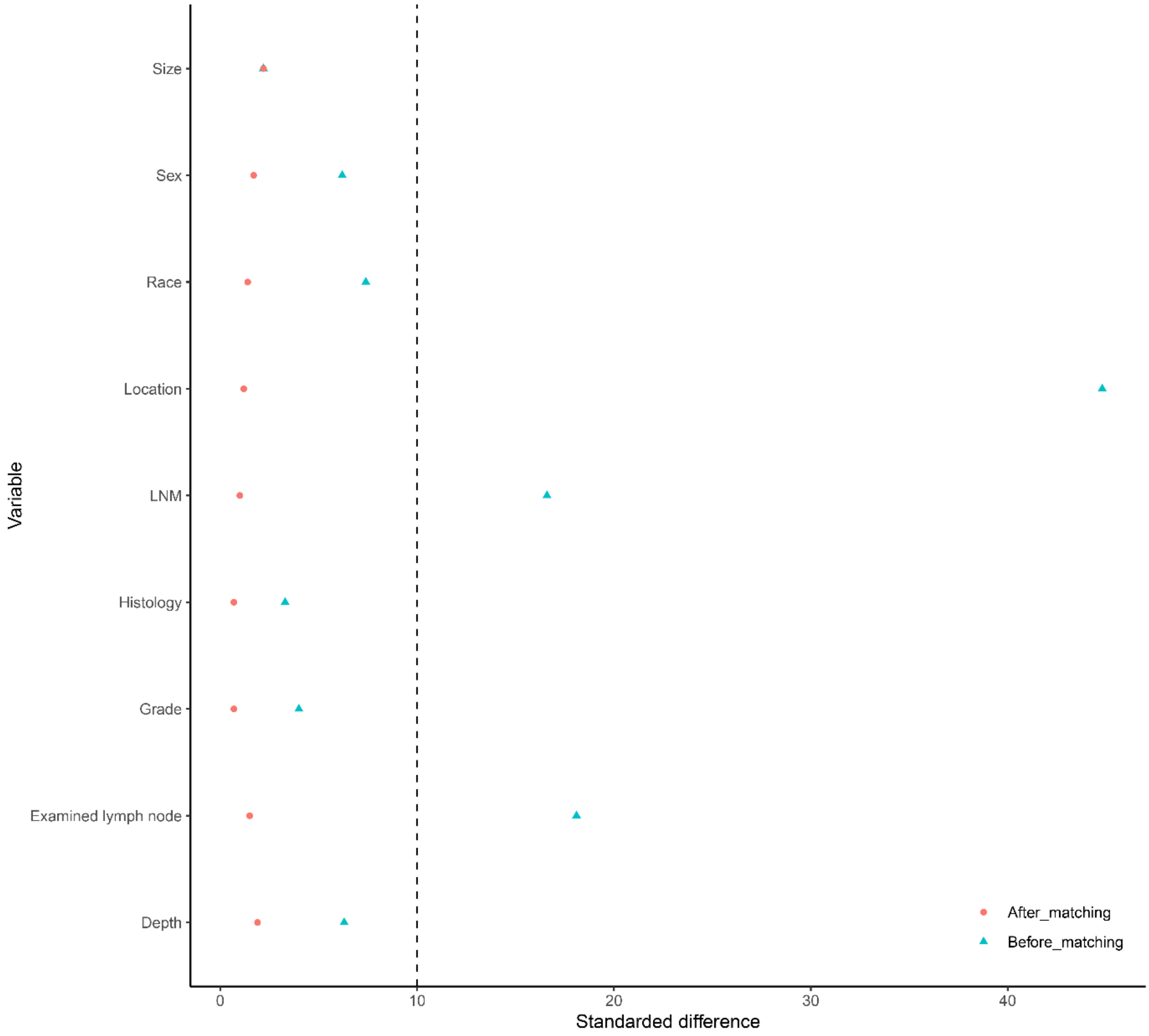

Supplementary Figure 8. Standardized difference across covariates before and after propensity score matching. The result showed that candidate covariates were well matched.
